# Unmasking the Hidden Risk of Systemic Toxicity from Topical Salicylates

**DOI:** 10.5811/westjem.46538

**Published:** 2025-09-01

**Authors:** Neelou Tabatabai, Swetaleena Dash, James A. Chenoweth, Timothy E. Albertson

**Affiliations:** * University of California, Davis, School of Medicine, Department of Emergency Medicine, Sacramento, California; † VA Northern California Health Care System, Medicine Department, Mather, California; ‡ University of California, Davis, School of Medicine, Department of Internal Medicine, Sacramento, California

## Abstract

**Introduction:**

Topical salicylates are commonly found in over-the-counter medications and are applied for pain relief or to treat dermatologic conditions. While generally considered safe, they can cause systemic toxicity under certain conditions. We conducted a systematic review of topical salicylate toxicity. This comprehensive review of previously reported cases highlights the risks, clinical presentations, and management considerations of systemic toxicity from topical salicylates.

**Methods:**

We present a new case of topical salicylate toxicity and conducted a comprehensive systematic literature search from 1952–2024 using PubMed, Google, and Google Scholar. Our search was supplemented by cross-referencing previous studies to identify cases and reviews of topical salicylate toxicity. We then performed a descriptive analysis of the cases, summarizing key information such as clinical presentation, blood levels, and outcomes. Findings were used to contextualize the risks and clinical manifestations of topical salicylate toxicity.

**Results:**

A total of 44 cases of topical salicylate toxicity, including our index case, were identified and included in our analysis. Most cases involved patients > 40 years of age, but all age ranges were represented, including neonates. The most frequently reported symptoms included tachypnea (32.5%) and vomiting (25.5%). The new case was an elderly male with further altered mental status from baseline dementia and elevated anion gap.

**Conclusion:**

Both the new case and the literature review emphasize the continued potential systemic risks of topical salicylates among a broad demographic. Given the variable presentations, clinicians should maintain a high index of suspicion for salicylate toxicity in patients with unexplained altered metabolic and/or mental status. Early consideration, recognition, and intervention of topical salicylates-induced toxicity is essential for good outcomes. As many of these products are heavily advertised, patient education on the appropriate use of topical salicylates may be crucial to prevent inadvertent toxicity.

## INTRODUCTION

Derived from willow bark (*Salix alba*), topical salicylates are used for both pain and dermatologic concerns. They are found in many common over-the-counter (OTC) products including aspirin, bismuth subsalicylate, and oil of wintergreen. When salicylates are applied to the skin, they work by acidifying the stratum corneum, promoting the desquamation of corneocytes, and reducing intercellular cohesion. This disruption weakens the skin’s barrier properties and facilitates topical absorption. Topical salicylates are available in a variety of formulations, including patches, gels, and creams, with concentrations ranging from 0.5% for cosmetic purposes to 50% for treating conditions such as warts.^1^ Many of these OTC products are heavily advertised by famous, former pro athletes without any risk discussion.

Despite their widespread use, topical salicylates can be absorbed enough to cause systemic toxicity particularly when a large dose of high concentration (milligrams per milliliter [mg/mL]) salicylates are applied to large body surface areas (amount applied) or with repeated applications (frequency). Individuals with risk factors such as decreased renal function or compromised skin integrity are at greater risk.^2^ However, toxicity can also occur in individuals without traditional risk factors highlighting the need for greater public education and clinical awareness about topical salicylate products. The condition of the skin, the concentration of the salicylate product, the vehicle/diluent used in the product, the frequency of application, and the surface area applied are all important variables that determine topical dosing and the potential for toxicity.

A recent case of an elderly male with altered mental status secondary to topical salicylate toxicity emphasizes that this remains a potential source of salicylate toxicity. The patient’s presentation demonstrates the potential for significant systemic absorption even in the absence of well-known risk factors such as damaged skin or significant renal impairment. Additionally, we review previously published case reports of topical salicylate toxicity to emphasize associated risks and the importance of early recognition, intervention, and prevention of misuse particularly in high-risk populations such as pediatric and elderly demographics. The dermal route of salicylates absorption, although rare, remains a clinical problem seen in the emergency department (ED).

## CASE REPORT

A 78-year-old male with achalasia type II, advanced dementia, alcoholic cirrhosis complicated by gastric varices, chronic heart failure with preserved ejection fraction, traumatic brain injury, and paroxysmal atrial fibrillation presented to the ED because of altered mental status over the prior three days. His family was concerned that he was less communicative and had a loss of appetite. He denied any symptoms of dizziness, lightheadedness, tinnitus, nausea, or vomiting. His medications included 12.5 mg of trazodone at night and 15 mL of lactulose twice daily. After a review of the chart and interviewing the family, there were no oral medications containing any formulations of salicylate in the household.

Initial vital signs included a blood pressure of 107/64 millimeters of mercury (mm Hg), heart rate of 85 beats per minute, and respiratory rate of 18 breaths per minute with an oxygen saturation of 98% on room air. The oral temperature was recorded as 36.9°C. The skin exam did not reveal any signs of lotions, patches, acute trauma, rashes, or infection. A basic metabolic panel revealed a creatinine of 1.02 mg per deciliter (mg/dL) similar to his prior values. Other initial lab work was notable for an elevated anion gap of 16 (sodium 141 millimoles (mmol) per dL; chloride 103 mmol/dL; carbon dioxide 22 mmol/dL; and potassium 4.0 mmol/dL); and a slightly elevated venous serum pH of 7.45. A comprehensive blood panel included a white blood cell count of 3.8 thousand cells per microliter (cells/μL), hemoglobin of 11.0 grams per dL (g/dL) and platelet count of 140 thousand cells/μL. Urinalysis found moderate bacteria, trace leukocyte esterase and trace ketones. An arterial blood gas found a partial pressure of oxygen of 100 mm Hg, 94% saturated, a partial pressure of carbon dioxide of 31 mm Hg, and a pH of 7.54 consistent with a respiratory alkalosis/alkalemia with mild hyperventilation. Imaging results, including a computed tomography of the head without contrast and a chest radiograph, were both unremarkable for any acute findings.

In evaluating the cause of his change in mental status and elevated anion gap, a serum salicylate level was drawn. The serum salicylate level was elevated to 41.4 mg/dL. His ethanol and acetaminophen levels were undetectable. When the family was told about the elevated salicylate level, they remembered that he had applied a three-ounce tube of ICY HOT pain-relieving cream (30% methyl salicylate and 10% menthol) to his back (estimated body surface area 18%) within a two-day period, prior to presentation to the ED. It is not known whether he washed his hands after applications. No occlusive dressings were used. The use instructions for the cream are vague and suggest applying a thin layer to the affected area and massage until thoroughly absorbed into the skin no more than 3–4 times a day. Washing hands after application is also suggested. Throughout his stay in the ED, his vital signs remained stable.

**Figure 1:**
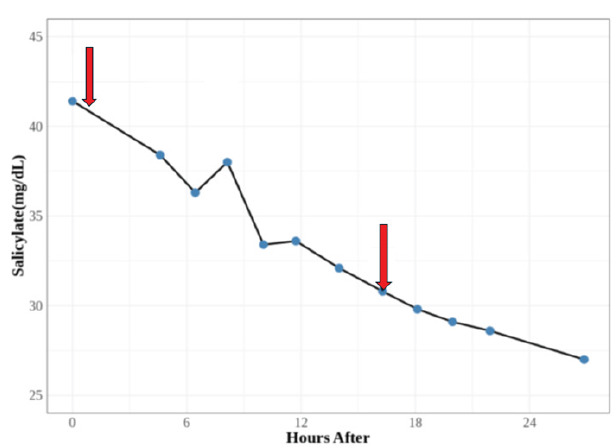
Trend of serum salicylate levels over two days. The arrows reflect the beginning and end of bicarbonate infusion. *mg/dL*, milligrams per deciliter.

A sodium bicarbonate infusion (3 amps of sodium bicarbonate in a liter of dextrose 5% in water after removing 100–150 mL, and infused at 100 mL per hour) with potassium chloride was started in the ED. The patient was admitted to the intensive care unit for close monitoring of symptoms, and frequent repeat serum salicylate levels, blood gases, and electrolyte values. Additional potassium was given to maintain the serum level ≥ 4 mmol/dL Although the patient denied symptoms of a urinary tract infection, given his urinalysis findings and altered mental state, he was empirically treated with antibiotics. The sodium bicarbonate infusion ran ≈ 12 hours and was stopped when the serum salicylate decreased below 30 mg/dL. The anion gap returned to normal. While the family was adamant that the only salicylates to which the patient had access were the topical products, we could not completely rule out additional oral or chronic poisoning. The patient was discharged after two days of hospitalization at his baseline mental status and with the last measured serum salicylate level of 27.0 mg/dL. Figure 1 summarizes the serum salicylate levels in this patient.

## METHODS

We performed a systematic and comprehensive review of cases of topical salicylate toxicity. A literature search using PubMed, Google, and Google Scholar identified relevant publications from 1952–2024. The search strategy employed the following combinations of terms: “topical salicylate toxicity,” “poisoning,” “salicylate,” “methyl salicylate,” and “case reports.” The search resulted in all available publications and specifically targeted peer-reviewed case reports, case series, conference abstracts, reviews, and letters to the editor. It also included texts found in Dutch, English, French, German, Italian, Polish, and Turkish. We used two previous literature reviews on topical salicylate toxicity published prior to our study to ensure all the papers were included. The first, by Brubacher and Hoffman in 1996, reviewed 17 cases.^3^ A subsequent review by Madan and Levitt in 2014 expanded the dataset to include 25 cases but did not include all the cases in the first review.^2^ The case data extractions were done by all the authors.

Some published literature was available only in abstract form. In those cases, we extracted all relevant information provided, such as the highest plasma salicylate concentrations and patient symptoms. However, if critical details such as patient outcomes were missing, this limitation was noted as “not available.” If no specific information about the salicylate poisoning could be obtained from an article or from the prior reviews, we excluded the article from our review. We also excluded five case reports due to lack of availability of the paper or incomplete details such as salicylate blood levels and case data.^4^–^8^ From each case report, we recorded the year of publication, patient age, sex, concentration of topical salicylate/vehicle, peak reported salicylate concentration (mg/dL), day of peak plasma levels, underlying diseases, case outcome, and physical exam findings. If available, we noted body weight, salicylate concentration, diluent, estimated surface exposure, length of exposure, and timing of administration. All salicylate units were converted into mg/dL for consistency. Intact skin was defined as having no compromised skin integrity secondary to underlying dermatological conditions.

## RESULTS

We found an additional 12 new cases of topical salicylate toxicity from published literature not included in the previous reviews. This expanded the total reported cases to 44, including the current case presented here (Table 1). The first review published in 1996 reviewed 17 cases.^3^ The 2014 review expanded the number of cases found to 25 but did not include all the cases in the first review.^2^ Seven were abstracts only.^9^–^15^ Of the 43 previously published case reports, eight were published in languages other than English.^9^–^11^,^16^–^21^

The cases reviewed included a wide age range; the youngest cases involved three newborns (< one month of age), and the oldest were two 80-year-olds.^3^,^26^,^29^,^32^,^34^ Demographic data showed that 67.4% (29/43) of cases occurred in males, 30.2% (13/43) in females, and one case (1/43) of no specified gender. Most cases occurred in patients > 40 years of age (18 cases). Children under one accounted for seven cases, while there were only four cases in children between 1–10 years of age. Two cases had no reported ages.

The lowest plasma concentration reported was 14.0 mg/dL, and the highest was 223.0 mg/dL.^21^,^33^ Two cases did not have recorded plasma concentrations, and one was sampled postmortem.^26^,^40^ The most common symptoms of salicylate poisoning noted included tachypnea (32.5%), vomiting (25.5%), nausea (21.0%), and tinnitus (21.0%). Of the 43 previously reported cases, the most common underlying medical conditions associated with topical toxicity were 17 patients with psoriasis, (35.5%) and 10 (23.2%) with ichthyosis. Seven cases involved toxicities with intact skin.^17^,^22^,^24^,^26^,^27^,^33^,^44^ Death occurred in five cases (11.6%). Death was observed in patients with psoriasis and tinea imbricata, and there was one isolated death reported in a patient with intact skin.^9^,^26^,^40^ The final outcome of seven cases was not available.^10^,^11^,^36^,^37^,^42^,^43^

## DISCUSSION

Our case report exemplifies the rare but important instance of systemic salicylate toxicity resulting from topical application of methyl salicylate over a large surface area in an elderly patient with multiple comorbidities. Because he applied the topical salicylates on himself, the amount that he may have ingested intentionally or accidentally is unknown. Since the last published reviews on topical salicylate poisonings, 13 cases have been added with 12 new cases reported from the literature and one case reported here.^2^,^3^ Despite reduced use of topical salicylate in dermatology, cases of topical salicylate toxicity continue to be reported. When informed of the blood salicylate levels, the family disclosed that the patient had recently been using a popular, aggressively marketed, methyl salicylate-containing cream with multiple applications to about 18% of his body surface area within a two-day period. Notably, despite intact skin and near-normal renal function, the patient had significant absorption of salicylate and a prolonged salicylate half-life resulting in toxicity.

In a voluntary study involving human subjects, the half-life after complete absorption of dermal salicylate was obtained from plasma concentrations. Subjects wore from 2–8 adhesive patches impregnated with methyl salicylate. The harmonic mean terminal half-life of methyl salicylate was determined to be 3.0 ± 1.2 hours.^4^ The half-life of methyl salicylate in our case report was prolonged and suggests continued absorption, additional oral exposure, or chronic salicylate exposure.

In cases where data were available, as in the case presented here, toxicities typically manifested within a few days of topical salicylate use. Severe toxicities, including seizures, severe metabolic acidosis, cerebral edema, acute respiratory distress syndrome, hypoglycemia, hypoglycorrhachia, ketosis, and death, were most often linked to applications of high-concentration salicylate products involving large body surface areas over extended periods. Elevated and toxic doses of salicylate typically occur with blood levels above 20–30 mg/dL, and can disrupt key physiological processes, leading to a wide range of harmful effects. Elevated toxic salicylate levels can impair oxidative phosphorylation, resulting in adenosine triphosphate production failure while also increasing oxygen consumption, metabolic acidosis, and heat generation.

Clinical manifestations in early toxicity often occur from stimulation of the brain’s respiratory center causing initial tachypnea and respiratory alkalosis, as seen in this case. The inhibition of Krebs cycle enzymes reduces glucose availability systemically and in the brain. This promotes organic acid accumulation, which leads to metabolic acidosis. Resultant significant fluid and electrolyte losses lead to dehydration, sodium and potassium depletion, and reduced buffering capacity. Once absorbed, salicylic acid is metabolized by the liver to more water-soluble byproducts.^5^ In considering topical dosing, the surface area treated, the concentration of salicylate used should not be excessive; the use of occlusive dressings, the frequency of application, and the patient’s underlying volume- and renal-status must be considered.^6^ A study with patients with active psoriasis found that after 10 hours with occlusive dressing over the topical salicylates, 60% of the salicylate was absorbed.^6^

The priority in treating any poisoned patient is assessing the airway, oxygenation, and maintaining perfusion of organs. Once toxicity is identified, it is critical to lower the salicylate levels. Resuscitation should begin with thorough skin decontamination with soap and water to remove any remaining drug and fluids, and dextrose 5% with 3 ampules of sodium bicarbonate to buffer the metabolic acidosis; added potassium chloride increases renal excretion and helps to prevent brain absorption by ion trapping of the salicylate. Potassium chloride is often added to prevent hypokalemia, the presence of which will inhibit alkalinization of the urine. Hemodialysis is indicated for severe acidosis, acute respiratory distress syndrome, severe altered mental status, seizures, kidney failure, refractory hypotension, and salicylate levels greater than 100 mg/dL for acute exposures and potentially lower levels for chronic exposure.^7^ The Extracorporeal Treatments in Poisoning workgroup recommendations and indications for salicylate poisoning are very useful.^45^

In addition to the concentration and surface area of application, different formulations of topical salicylic acid have different levels of systemic absorption. A study by Morra et al found that the methyl salicylate used in the current case demonstrated higher absorption compared to other formulations such as trolamine salicylate.^8^ Methyl salicylate reached detectable serum salicylate levels within one hour of application with cumulative absorption increasing after repeated applications. In contrast, trolamine salicylate showed minimal skin absorption, with serum salicylate levels often undetectable and significantly lower recovery in urine.^8^ Our review of 43 previous cases plus the current case of topical salicylate toxicity corroborates this observation.

As summarized in Table 1, symptoms of topical salicylate toxicity are broad and generally develop within a few days of use, particularly when applied over large body surface areas, compromised skin, or with repeated applications. While the most common findings in the literature review were metabolic abnormalities, altered mental status, tachypnea, vomiting, nausea, and tinnitus, the current patient had altered mental status, metabolic acidosis with elevated anion gap, and tachypnea (with respiratory alkalemia) but only reported tinnitus after he regained his baseline mental status and the salicylate levels had fallen. This suggests that older patients or those with comorbid conditions like dementia can be a challenge to diagnose and demonstrates the need for heightened clinical suspicion when presented with a limited history and symptoms.

This case report and descriptive analysis of similar historical cases emphasizes the need for further patient education about the potential toxicity of OTC topical salicylates and their use to improve their safety among the general population and particularly in vulnerable populations such as children, renal-compromised patients, and patients with baseline altered mental status. This report reinforces the need for clinicians to suspect and inquire about topical salicylate use in patients with an elevated anion gap and altered mental status given its widespread use and availability.

## LIMITATIONS

Retrospective literature research has several limitations. Some older publications, particularly those predating digital archiving, may have been excluded in the search due to their limited availability and not being included in the electronic databases searched. A few publications were not available. If available, abstracts were used, but some provided limited data such as patient outcomes, sex, age, and symptoms. Publication bias clearly favors publication of toxic cases but considering the limited number of cases found over 72 years, the incidence of topical salicylate toxicity must be low. Case reports have significant limitations. This case report also has limitations in terms of history and potential chronic or oral dosing of salicylates that was denied by the family but still could have happened.

## CONCLUSION

Topical salicylates have the potential for systemic toxicity particularly when large doses of methyl salicylate are applied in high concentrations in vulnerable populations such as patients with compromised renal function, and in pediatric patients with a large surface-to-body ratio. The inappropriate use of methyl salicylate products in particular, especially in high concentrations over large body surface areas, on compromised skin, and with repeated applications can pose significant health risks. Clinicians should maintain a high index of suspicion for salicylate toxicity in patients presenting with non-specific symptoms such as altered mental status, or unexplained metabolic acidosis or tachypnea especially in those with a history of topical salicylate use. Although salicylate toxicity is not common, early recognition and intervention are essential to prevent or reduce clinical toxicity and ensure rapid resolution. Widespread patient education about the toxicological risk of these heavily advertised topical salicylate products is also needed.

## Figures and Tables

**Table 1:** Cases of toxicity from topical salicylic acid: 1952 to present.

Year published	Age (years)	Sex	Formulation/concentration/Vehicle (if available)	Peak plasma mg/dL	Day	Underlying disease	Outcome	Symptoms	Reference
2024	50 days	M	magistral salicylate cream	46.9	n/a	Scabies	R	reduced sucking, coughing, tachypnea	16 *
2021	78	F	pure salicylic acid powder	79.8	n/a	Schizoaffective Disorder, Scabies Delusion	R	subacute confusion, tinnitus, weakness, double vision	22
2016	14	M	60-gram tube of 30% methyl salicylate ointment; masturbation	68.0	1	None	R	shortness of breath, chest pain, lightheadedness, vomiting, malaise, tachypnea	
2015	2 months	M	50% salicylic acid; scalp	42.5	2	Crusta Lactea	R	fever, breathlessness, irritability	23
2015	54	M	methyl salicylate ointment; 1 tube per day on back for 7 days	78.6	7	Asthma, Obstructive Sleep Apnea, Type II Diabetes Mellitus, Hypertension, Hypercholesteremia	R	shortness of breath, tachypnea	24
2015	54	M	26% methyl salicylate gel; 1 tube daily for 7 days	78.7	7	Type II Diabetes Mellitus, Hypertension	R	nausea, vertigo, tachypnea	17
2012	6 weeks old	n/a	23% salicylic acid; scalp	58.0	3	Seborrheic Dermatitis	R	tachypnea, impaired consciousness	25 *
2007	80	M	35% methyl salicylate oil, on lower extremities regularly (accidentally drank a mouthful of oil)	82.6 (pm)	1	Type II Diabetes Mellitus, End-stage Renal Disease, Coronary Artery Disease	D	seizures, unresponsive, apnea	26
2005	27	M	20% salicylic acid; arms, legs, abdomen; approximately 56% BSA	60.0	1	None	R	tinnitus, malaise, nausea	27
2003	58	M	10% salicylate acid ointment; greater than 80% BSA	43.5	5	Psoriasis	D	n/a	9 *
2003	35	M	10% salicylate acid ointment; greater than 80% BSA	51.0	4	Psoriasis	D	n/a	9 *
2002	40	M	Total body application of a “yellow cream”	48.5	1	Psoriasis	R	tachypnea, vomiting, tinnitus, drowsy, flushed, sweaty	28
2002	Newborn	M	20% salicylate petroleum ointment; 2 times daily over the whole body	119.0	7	Ichthyosis	R	tachypnea, renal failure, heart failure	29
2002	31	M	30% salicylic ointment; back and upper and lower extremities continuously	30.9	2	Psoriasis, HIV	R	tachypnea, altered mental status	30
1999	70	M	salicylic acid cream	69.5	5	Psoriasis	R	encephalopathy	18 *
1997	36	F	10% salicylate acid ointment	30.0	4	Psoriasis	n/a	fever, deafness	10 *
1997	5	F	10% salicylate acid ointment; applied 3 times within 36 hours on the whole body	29.1	2	Lamellar Ichthyosis	R	hyperpnea, lethargy, altered mental status, nausea, vomiting, fever, hypotonic limbs, oculogyric crisis	31
1996	80	F	2%–10% salicylic acid ointment; body and scalp	46.5	6	Erythroderma	R	Confused, drowsy, paranoid, deep respirations, tachypnea	3
1996	7	M	10% salicylic acid ointment; large surface area of body over a period of 4 weeks	98.5	28	Ichthyosis Vulgaris	R	vomiting, tinnitus, vertigo, wheezing, hyperventilation, deep somnolence	19 *
1994	3 months	F	4% salicylate ointment	55.5	2	Ichthyosis	R	febrile, diarrhea, vomiting, convulsions, apnea	20
1994	79	M	2%–5% salicylic acid with crude coal tar	45.0	7	Psoriasis, Hypertension, Type II Diabetes Mellitus, Atrial Fibrillation, Acute Renal Failure	R	Unresponsive, hypoglycemia	12
1994	42	F	10% salicylic acid; 50 grams to trunk and limbs daily	36.0	10	Psoriasis	R	agitation, fever, deafness, nausea	13
1992	27	M	40% salicylic acid ointment; applied once to 41% BSA	83.6	1	Psoriasis	R	nausea, vomiting, hyperthermia	14
1991	72	M	10% salicylic acid cream; 80% BSA for 3–4 weeks	44.3	21–28	Psoriasis, End-stage Renal Disease, Type II Diabetes Mellitus	R	confusion, nausea, weakness, tachypnea	15
1990	Newborn	M	2% salicylic acid cream; applied every 3–4 hours	42.9	3	Collodion-like membrane	R	vomiting	32
1990	12	M	2%–10% salicylic acid cream; applied to the whole body 2 times per day	45.7	8	Ichthyosis	R	salicylate toxicity	32
1989	61	F	methyl salicylate ointment on knees	34.5	14	Osteoarthritis, Mitral Stenosis	R	bruising	33
1989	Newborn	F	1% salicylic acid; every 3 hours to whole body	58.7	1	Harlequin Fetus	R	tachypnea, fever	34
1986	45	M	3% salicylic in crude coal tar; 3 times per day to body below the neck	25.2	5	Psoriasis, Psoriatic Arthritis	R	tinnitus	35
1980	72	M	5% salicylic acid; 8 times per day	14.0	7	Ichthyosis, Renal Failure	n/a	salicylate toxicity (Metabolic acidosis)	36
1980	48	M	20% salicylic acid in petrolatum	81.0	6	Psoriasis	n/a	coma	11 *
1979	42	M	40% salicylic acid ointment; applied to hands and feet; 2% salicylic acid ointment was applied to rest of body	73.0	1	Psoriasis	n/a	sweating, flushing, deafness	37
1979	20	F	15% salicylic acid; 1 time application	71.0	1	Ichthyosis	n/a	abdominal pain, tinnitus	37
1979	30	M	4%–12% salicylic acid; applied 2 times per day to trunk and limbs	45. 5	20	Ichthyosis	R	malaise, nausea, tinnitus, deafness, epigastric discomfort, hyperventilating	38
1978	12	M	2%–10% salicylic acid cream; applied to body 2 times per day	46.0	3	Ichthyosis	R	salicylate toxicity	39
1975	62	F	10% salicylic acid ointment; 75% BSA	223.4	18	Psoriasis	R	dry mouth, headache, tinnitus	21 *
1968	n/a	M	20.7% salicylic acid solution; 50% BSA 2 times per day	n/a	1	Tinea Imbricata	D	tachypnea, fever, comatose	40
1968	n/a	M	20.7% salicylic acid solution; 50% BSA 2 times per day	n/a	1	Tinea Imbricata	D	tachypnea, fever, ataxia, altered mental status, comatose	40
1964	39	F	6% salicylic acid; 6 times per day	64.0	11	Psoriasis	R	dyspnea, nausea, vomiting, headache, dizziness, tinnitus	41
1964	47	F	3% salicylic acid; 6 times per day	46.0	7	Psoriasis	R	dyspnea, nausea, thirst, headache, dizziness, agitation, hallucinations, fever	41
1964	55	M	6% salicylic acid; 6 times per day	47.0	4	Psoriasis	R	anorexia, dizziness, vomiting, agitation	41
1953	6	M	10% salicylate acid; 3 times per day	46.0	2	Ichthyosis	n/a	abdominal pain, vomiting, tachypnea, irritability	42
1952	10	F	3% salicylate acid ointment; 6 times per day	40.0	5	Ichthyosis	n/a	abdominal pain, vomiting, lethargy, hallucinations	43

* Published in a non-English language.

** Salicylate toxicity was the only recorded symptom/effect.

*R*, resolved; *D*, death; *F*, female; *M*, male; *n/a*, not available; *pm*, postmortem; *BSA*, body surface area, *newborn*, < 1 month of age.

## References

[1] Yeoh SC, Goh CF (2022). Topical delivery of salicylates. Drug Deliv Transl Res.

[2] Madan RK, Levitt J (2014). A review of toxicity from topical salicylic acid preparations. J Am Acad Dermatol.

[3] Brubacher JR, Hoffman RS (1996). Salicylism from topical salicylates: review of the literature. J Toxicol Clin Toxicol.

[4] Martin D, Valdez J, Boren J (2004). Dermal absorption of camphor, menthol, and methyl salicylate in humans. J Clin Pharmacol.

[5] Temple AR (1978). Pathophysiology of aspirin overdosage toxicity, with implications for management. Pediatrics.

[6] Taylor JR, Halprin KM (1975). Percutaneous absorption of salicylic acid. Arch Dermatol.

[7] Runde TJ, Nappe TM, Okocha, Shumway (2023). Salicylates toxicity. StatPearls.

[8] Morra P, Bartle WR, Walker SE (1996). Serum concentrations of salicylic acid following topically applied salicylate derivatives. Ann Pharmacother.

[9] Chodorowski Z, Anand JS, Waldman W (2003). [Suicidal salicylate intoxications and unintentional percutaneous poisoning with salicylic ointment]. Przegl Lek.

[10] Jongevos SF, Prens EP, Wolterbeek JH (1997). [Acute perceptive hearing loss and metabolic acidosis as complications of the topical treatment of psoriasis with salicylic acid-containing ointment]. Ned Tijdschr Geneeskd.

[11] Treguer H, Le Bihan G, Coloignier M (1980). [Salicylate poisoning by local application of 20% salicylic acid petrolatum to a psoriatic patient]. Nouv Presse Med.

[12] Maurer TA, Winter ME, Koo J (1994). Refractory hypoglycemia: a complication of topical salicylate therapy. Arch Dermatol.

[13] Dwyer CM, McCloskey RH, Kerr RE (1994). Poisoning from topical salicylic acid. Postgrad Med J.

[14] Pec J, Strmenova M, Palencarova E (1992). Salicylate intoxication after use of topical salicylic acid ointment by a patient with psoriasis. Cutis.

[15] Raschke R, Arnold-Capell PA, Richeson R (1991). Refractory hypoglycemia secondary to topical salicylate intoxication. Arch Intern Med.

[16] Fil E, Dilek S, Umur O (2024). An infant developed intoxication following topical salicylate use: a case report. Ped Acad Case Report J.

[17] Wong A, Mac K, Aneman A (2016). Modern intermittent haemodialysis (IHD) is an effective method of removing salicylate in chronic topical salicylate toxicity. J Med Toxicol.

[18] Pertoldi F, D’Orlando L, Mercante WP (1999). [Acute salicylate intoxication after trancutaneous absorption]. Minerva Anestesiol.

[19] Germann R, Schindera I, Kuch M (1996). [Life-threatening salicylate poisoning caused by percutaneous absorption in severe ichthyosis vulgaris]. Hautarzt.

[20] Abdel-Magid EH, el-Awad Ahmed FR (1994). Salicylate intoxication in an infant with ichthyosis transmitted through skin ointment--a case report. Pediatrics.

[21] Luderschmidt C, Plewig G (1975). [Chronic percutaneous salicylic acid poisoning]. Hautarzt.

[22] Renzetti M, Schenck LEE, Mailman J (2021). Slathering salicylates: a topical story. Am J Respir Crit Care Med.

[23] Vazquez Martinez JL, Stanescu S, Castrillo Bustamante S (2015). Unrecognized transcutaneous severe salicylate intoxication in an infant. Pediatr Emerg Care.

[24] Robinson K, Rauch A, Hannan L (2015). Salicylate poisoning following topical administration of methylsalicylate. Emerg Med Australas.

[25] Oualha M, Dupic L, Bastian C (2012). [Local salicylate transcutaneous absorption: an unrecognized risk of severe intoxication: a case report]. Arch Pediatr.

[26] Chin RL, Olson KR, Dempsey D (2007). Salicylate toxicity from ingestion and continued dermal absorption. Cal J Emerg Med.

[27] Cull M, Vicas IM-O (2005). Salicylate Toxicity with Topical Exposures on Intact Skin. Abstracts of the 2005 North American Congress of Clinical Toxicology. Clin Toxicol.

[28] Bell AJ, Duggin G (2002). Acute methyl salicylate toxicity complicating herbal skin treatment for psoriasis. Emerg Med (Fremantle).

[29] Yamamura S, Kinoshita Y, Kitamura N (2002). Neonatal salicylate poisoning during the treatment of a collodion baby. Clin Pediatr (Phila).

[30] Peyriere H, Balmes N, Rouanet I (2002). Acute salicylate intoxication after percutaneous absorption in an HIV patient treated for psoriasis. AIDS.

[31] Chiaretti A, Schembri Wismayer D, Tortorolo L (1997). Salicylate intoxication using a skin ointment. Acta Paediatr.

[32] Galea P, Goel KM (1990). Salicylate poisoning in dermatological treatment. Arch Dis Child.

[33] Chow WH, Cheung KL, Ling HM (1989). Potentiation of warfarin anticoagulation by topical methylsalicylate ointment. J R Soc Med.

[34] Ward PS, Jones RD (1989). Successful treatment of a harlequin fetus. Arch Dis Child.

[35] Shupp DL, Schroeter AL (1986). An unusual case of salicylate toxicity. J Am Acad Dermatol.

[36] Smith WO, Lyons D (1980). Metabolic acidosis associated with percutaneous absorption of salicylic acid. J Okla State Med Assoc.

[37] Anderson JA, Ead RD (1979). Percutaneous salicylate poisoning. Clin Exp Dermatol.

[38] Davies MG, Briffa DV, Greaves MW (1979). Systemic toxicity from topically applied salicylic acid. Br Med J.

[39] Aspinall JB, Goel KM (1978). Salicylate poisoning in dermatological therapy. Br Med J.

[40] Lindsey CP (1968). Two cases of fatal salicylate poisoning after topical application of an antifungal solution. Med J Aust.

[41] Vonweiss JF, Lever WF (1964). Percutaneous salicylic acid intoxication in psoriasis. Arch Dermatol.

[42] Cawley EP, Wheeler CE (1953). Salicylic acid poisoning in dermatological therapy. J Am Med Assoc.

[43] Young CJ (1952). Salicylate intoxication from cutaneous absorption of salicylic acid. South Med J.

[44] Thompson TM, Toerne T, Erickson TB (2016). Salicylate toxicity from genital exposure to a methylsalicylate-containing rubefacient. West J Emerg Med.

[45] Juurlink DN, Gosselin S, Kielstein JT (2015). Extracorporeal treatment for salicylate poisoning: systematic review and recommendations from the EXTRIP Workgroup. Ann Emerg Med.

